# Barriers to and Facilitators of the Consumption of Animal-Based Protein-Rich Foods in Older Adults: Re-Analysis with a Focus on Sustainability

**DOI:** 10.3390/nu15020470

**Published:** 2023-01-16

**Authors:** Katherine M. Appleton

**Affiliations:** Research Centre for Behaviour Change, Department of Psychology, Faculty of Science and Technology, Bournemouth University, Poole House, Fern Barrow, Poole BH12 5BB, UK; k.appleton@bournemouth.ac.uk

**Keywords:** protein, older adult, taste, enjoyment, convenience, questionnaire

## Abstract

Older adults may gain health benefits from the consumption of animal-based protein-rich foods, but environmental pressures suggest advocating some meat and dairy foods over others, and understanding the barriers and facilitators for consuming these different foods would be of value. Existing data on the barriers to and facilitators of the consumption of meat and dairy products were re-analysed for differing effects for white, red, and processed meat consumption and for yoghurt, soft cheese, and hard cheese consumption. White meat consumption was associated with fewer concerns over spoilage and waste and stronger perceptions that meat is convenient (smallest Beta = 0.135, *p* = 0.01), while red and processed meat consumption were positively associated with liking /taste, appearance, and convenience (smallest Beta = 0.117, *p* = 0.03). Yoghurt and soft cheese consumption were positively associated with liking/taste and medical concerns, and fewer concerns over ability and habit (smallest Beta = −0.111, *p* = 0.05), while hard cheese consumption was only associated with liking/taste (Beta = 0.153, *p* = 0.01). Taken together, these data suggest that enhancing or promoting the enjoyment, taste, visual appeal, and ease-of-consumption of the more sustainable meat and dairy options may be of value in encouraging the consumption of these foods in older adults.

## 1. Introduction

Protein consumption in older adults is important. Protein status tends to reduce with age as a result of age-related declines in protein absorption, metabolism and disposal, coupled with declines in protein intake [1,2,3]. This low protein status is associated with reduced muscle mass and size, reduced bone health and immune function, which result in an increased risk of falls, fractures, infection, morbidity, and mortality [1,2,3,4]. Adequate protein consumption can reduce or mitigate these concerns, such that several select committees and researchers recommend protein intakes for older adults of at least 1.0–1.2 g protein/kg body-weight/day, compared to the lower consumption of 0.8 g/kg/day for healthy younger adults [1,2,3]. Some evidence also suggests additional benefits from the consumption specifically of proteins from animal sources for older adults [4,5,6,7] due to the high essential amino acid content of these proteins [5,6,7] and associations between the consumption of animal-based proteins and positive health outcomes in this population group [4,6,7,8,9,10].

Alongside these recommendations, current sustainability concerns are also driving a need and desire for the reduced consumption of animal-based foods [11,12,13]. Some animal-based protein sources, however, have a lower environmental impact than others. The consumption of white meat (from poultry) is estimated to have an environmental impact that is lower than that of the consumption of red meat (from pigs, cattle, and sheep) and processed meats [12,14,15]. The production of hard cheeses can also have high environmental impacts, while the production of softer dairy products, such as yoghurts and milk, has lower estimates [14,15]. The consumption of white meats can also be recommended for health reasons compared to the consumption of red and processed meats [12,16,17,18,19,20], and, while increasing evidence suggests health benefits from the consumption of all dairy foods [21,22,23], greater benefits from softer compared to harder dairy products are reported in some studies [21,22]. Softer dairy foods may also be of benefit specifically for older adults due to their high water content and softer texture, considering increased concerns over hydration, dentition and physical abilities in this population [24]. Thus, both environmental and health considerations would suggest benefits from the consumption of white meats and softer dairy products for older adults. 

Understanding why individuals do or do not consume certain foods is the first step to encouraging their consumption. Repeated work has investigated the factors determining protein consumption and the consumption of protein-rich foods in older adults [25,26,27,28]. This work finds consumption to be largely influenced by a food’s sensory aspects, such as taste and pleasure; the practical aspects of consumption, including hard textures, difficulties with cutting, preparing and cooking, and difficulties with digestion; and changes to living situation, such as increased loneliness, isolation and financial concerns [25,26,27,28,29], although the relative importance of some factors compared to others for the consumption of different protein-rich foods has also been found [25,26]. 

Using data from an existing data set, this analysis investigated the barriers to and facilitators of the consumption of white meat, red meat, processed meats, yoghurts, soft cheeses, and hard cheeses in older adults. 

## 2. Materials and Methods

### 2.1. Existing Data Set

The existing data set comprised data from 351 UK older adults collected by questionnaire. Full details of the methodology for the original study are given in our earlier publication [25]. Briefly, one thousand older adults were sent a postal questionnaire from June 2013–January 2015. Adults were required to be aged 65 years or over, living in their own homes, in the UK, and content to be contacted. No other inclusion or exclusion criteria were applied to maintain the generalizability of the sample. Ethical approval for the study was gained from the Research Ethics Committee of Bournemouth University, UK (ID: 931), and all participants provided written informed consent in advance of participation. 

The questionnaire assessed intakes of various animal-based protein-rich foods, barriers and facilitators associated with intake, and various demographic and lifestyle characteristics. Protein-rich foods included meat, fish, eggs, and dairy products, and intakes were queried via a food frequency format asking for frequency of consumption, converted to provide the frequency of consumption per day. For the purposes of the current analysis, white meats (e.g., chicken, turkey), red meats (e.g., beef, lamb, pork), and processed meats (e.g., ham, bacon, sausages) were queried separately, as were yoghurts, custards and blancmanges, soft cheeses (e.g., cream cheese, Dairylea, camembert), and hard cheeses (e.g., cheddar, stilton, Emmental). Barriers and facilitators were investigated using 38 attitudinal statements per food group, which related to 19 potential barriers and facilitators as gained from an earlier focus group study [26]. Statements were responded to on a 7-point scale, from strongly disagree to strongly agree, and scored to result in a score per barrier or facilitator where higher scores denoted higher agreement that the barrier/facilitator was important to consumption. Demographic and lifestyle characteristics assessed were gender, age, marital status, living status, area of residence in the UK, Index of Multiple Deprivation (IMD) for the residential postcode, years of education, nationality, body mass index (BMI), denture-wearing, presence of physical disabilities that may hinder food purchasing, preparation, or consumption, and frequency with which older adults received help with food shopping, cooking, had food delivered, or ate away from their home. Season of questionnaire completion (summer/winter) was also recorded. The sample size was calculated based on the number of variables to be included in analyses (19 barriers/facilitators and 13 demographic/lifestyle variables), assuming a response rate of 30–35%.

The current analyses differ from our original analyses of this data set through the completion of separate analyses for each food group within the ‘meat’ and ‘dairy’ food categories to enable consideration of sustainability concerns. In the original investigation, analyses were conducted on the barriers and facilitators associated with ‘meat’ consumption, a composite variable composed of ‘red meats’, ‘white meats’ and ‘processed meats’, while in the current analyses, barriers to and facilitators of each of these meat categories were investigated separately. Similarly, for dairy consumption, analyses were originally conducted on the barriers to and facilitators of ‘dairy’ consumption, a composite measure of ‘yoghurts, custards and blancmanges’, ‘soft cheeses’ and ‘hard cheeses’, while the current analyses treat each of these product categories separately.

### 2.2. Analyses

Associations between intake frequency for each of the separate food categories, barriers and facilitators for each food group and demographic and lifestyle variables were investigated using multiple linear regression. Correlational analyses prior to these regression analyses revealed high correlations between marital status and living status; thus, only living status was used in all models. High correlations in the barriers to and facilitators of meat consumption were also found between barriers ‘liking’ and ‘taste’, and barriers ‘living alone’ and ‘smell’. To avoid issues due to multi-co-linearity, only data for ‘liking’ and ‘living alone’ were used. In the barriers and facilitators for dairy consumption, high correlations were found between ‘liking’ and ‘taste’, between ‘living alone’ and ‘smell’, and between ‘fresh’ and ‘quality’. To avoid concerns in the analyses on dairy consumption, only barriers ‘liking’, ‘living alone’, and ‘fresh’ were used. Nationality was not included in regression models but was instead checked to ensure a predominantly UK sample. Regression models were conducted using a stepwise method to predict the intake frequency of each food category using all available variables. 

## 3. Results

Complete questionnaire responses were gained from 351 (35.1%) individuals. Full details of the dataset are provided in our original publication [25]. Briefly, the dataset was composed of 149 (42%) males, 202 (58%) females; 95 (27%) aged 65–69 years, 90 (26%) aged 70–74 years, 70 (20%) aged 75–79 years, 49 (14%) aged 80–84 years, 17 (5%) aged 85–89 years, and three (1%) aged 90–94 years. Of these, 208 individuals (60%) lived with others, 133 (38%) lived alone, 146 (42%) lived in the South of the UK, 89 (25%) lived in the Midlands and Wales, 67 (19%) lived in the North of England, 45 (13%) lived in Scotland and Northern Ireland, and all, except 10 individuals, reported their nationality as English, Welsh, Scots, Northern Irish, or British. The sample was representative of the UK older population based on the 2011 census in terms of gender (χ^2^(1) = 0.29, *p* > 0.05) and age (χ^2^(3) = 4.90, *p* > 0.05), but more individuals resided in the South of the UK than in the census (χ^2^(3) = 18.10, *p* < 0.05). 

Individuals had 8 to 24 years of education (mean = 13 (SD = 3) years), a BMI of 16–52 kg/m^2^ (mean = 31 (SD = 5) kg/m^2^), and scores for deprivation (IMD) ranged from 0.01 to 0.99, with a mean of 0.60 (SD = 0.24). The sample was generally able (185 (53%) individuals did not wear dentures, 111 (31%) wore partial dentures, 55 (16%) individuals wore full dentures; 287 (82%) individuals reported no disabilities that hindered food purchasing, preparation or consumption; 243 (69%) individuals did not receive help with food shopping, cooking, or have food delivered), but the full range of disabilities was also reported. The majority of individuals (68%) ate out once a month, 62 (18%) ate out at least once a week, while 126 (32%) individuals never ate out. 

### 3.1. Meats

A total of 340 individuals reported consuming meats and completed this section of the questionnaire. White meats were consumed from never (18 participants) to every day (3 participants), where the majority of participants (173 participants) consumed white meats 0.2 times/day (or the equivalent of once every 4–5 days), and the mean (SD) for the whole sample was 0.24 (0.2) times/day. Red meats were consumed from never (41 participants) to every day (3 participants), where the majority of participants (154 participants) consumed red meats 0.2 times/day, and the mean (SD) for the sample was 0.22 (0.2) times/day. Processed meats were consumed from never (50 participants) to every day (5 participants), where the majority of participants (127 participants) consumed processed meats 0.2 times/day, and the mean (SD) for the sample was 0.19 (0.2) times/day.

Results of the regression analyses for the consumption of each category of meat are given in Table 1. 

Individuals who consumed white meats more frequently were younger and with fewer years of education. Greater consumption was associated with lower concerns over spoilage and waste and stronger perceptions that meat is convenient. Individuals who consumed red meats more frequently were more likely to be female and have fewer disabilities. Greater consumption was associated with a higher liking or appreciation of the taste of meat. Individuals who consumed processed meats more frequently were more likely to live with others. Greater consumption was associated with a higher liking/appreciation for the taste of meat, greater importance attached to the appearance of meat, lower concerns over the origins of meat, and stronger perceptions that meat is convenient.

### 3.2. Diary Products

A total of 347 individuals reported consuming dairy products and completed this section of the questionnaire. Yoghurts, custards, and blancmanges were consumed from never (73 participants) to every day (82 participants, the majority), and the mean (SD) for the whole sample was 0.41 (0.4) times/day (or the equivalent of once every 2–3 days). Soft cheeses were consumed from never (138 participants, the majority) to every day (5 participants), and the mean (SD) for the sample was 0.14 (0.2) times/day. Hard cheeses were consumed from never (53 participants) to every day (13 participants), where the majority of participants (106 participants) consumed hard cheeses 0.2 times/day, and the mean (SD) for the sample was 0.27 (0.3) times/day.

Results of the regression analyses for the consumption of each category of dairy product are given in Table 2. Individuals who consumed yoghurts, custards and blancmanges more frequently were more likely to be female. Greater consumption was associated with a higher liking for or a greater appreciation of the taste of dairy products. Individuals who consumed soft cheeses more frequently wore fewer dentures. Greater consumption was associated with a higher liking for/appreciation of the taste of dairy, fewer concerns over ability, weaker habits/less reliance on upbringing, and more agreement with consuming dairy for medical reasons. Individuals who consumed hard cheeses more frequently were more likely to be male and have more years of education. Greater consumption was associated with a higher liking/appreciation of the taste of dairy products. 

## 4. Discussion

The work was conducted to understand the barriers and facilitators, in older adults, to the consumption of white meats, red meats, processed meats, yoghurts, soft cheeses, and hard cheeses, with a view to encouraging the consumption of more sustainable animal-based protein-rich foods in this population group. Associations between consumption, several personal characteristics and a number of barriers and facilitators were found, such that suggestions for changing intakes can be made. 

White meats were consumed more frequently than red or processed meats, a preference between meat options that was recently demonstrated in older adults elsewhere [30]. White meats were more frequently consumed by individuals who were younger, with fewer years of education, and by those with fewer concerns over spoilage and waste, and stronger perceptions that meat is convenient. Red meats were more frequently consumed by females, those with fewer disabilities, and by those who reported stronger liking or perceptions that meat is tasty. Processed meats were more frequently consumed by those who live with others compared to those living alone and by those who reported stronger liking or perceptions that meat is tasty, who attach greater importance to the appearance of meat, who are less concerned with the origins of meat, and who consider meat to be convenient. 

With a focus on the barriers and facilitators, greater white meat consumption was associated with fewer concerns over spoilage and waste, and stronger perceptions that meat is convenient. These findings likely arise from the simple and easy methods by which often pre-prepared white meats can be cooked and eaten, which also results in little waste. Traditional stock- and soup-making from bones and carcasses will also result in low waste, activities more likely to be undertaken by the older population than a younger one. These findings suggest that focus on the use of pre-prepared and/or pre-cooked white meats and on simple and easy methods for preparation, cooking and eating may encourage increased white meat consumption. The importance of ease and low effort, as aspects of convenience, in preparing and consuming foods has previously been identified for older adults [25,26], and suggestions for increasing consumption by increasing the ease and reducing the effort involved have previously been made [30,31,32,33,34,35]. 

Red and processed meats were more frequently consumed for reasons of liking and taste. Other studies also demonstrate the importance of taste in meat consumption across the adult age range [36,37], including a greater importance for red meat than white meat consumption [37]. These findings may suggest that methods to enhance the taste of white meats or increase liking for dishes based on white meats would be of value. Taste and liking are known to be important predictors of food intake in older adults [25,26,27,29,30,38,39,40], and while taste perception is known to deteriorate with age [38,39,40,41], various studies demonstrate increased intakes following the use of flavour enhancers or added flavours in older adults [42,43,44,45,46,47,48,49]. Some consideration for individual preferences, however, is also required; work suggests that not all individuals respond to flavours with increased consumption [38,40,41,43,48,49]. Flavour use varies widely in the older population [29], as do flavour preferences [27,29,30,36,38,40,41,50], although consistent preferences for flavours that are perceived to be natural, appropriate to the meal, occasion, or culture are found [29,51,52,53]. Suggestions to enhance liking for the taste of white meats and white meat dishes may benefit from consideration of foods that are naturally flavoursome, such as lemon, ginger, herbs, and fruits [46,50,54], established flavour combinations, e.g., turkey and cranberry [51,52,53], and the use of self-serving, to allow individuals to accommodate personal preferences [44,45,49]. 

Processed meats were also more likely to be consumed by those who value appearance. Strategies to increase the visual appeal of white meats may be of value. Visual appeal is known to be important for food intake [26,27,38,49,50] not only in terms of attractiveness but also to allow individuals to recognize foods [38,55], expect certain flavours [38,55], and make assessments of quality and freshness [36]; aspects of meat that are known to be important to older adults [26] and that are predicting increasing white meat consumption trends [30,56]. Limited work to date, however, has investigated the importance of visual cues for older adults [38], and particular benefit may be gained from considering many senses alongside each other [38,41,49]. Work to establish how white meats and white meat dishes could look as well as taste more appealing to older adults would be of interest.

Associations with the demographic and lifestyle variables would also suggest benefits from strategies that focus on enhancing taste, appearance and easing the practical aspects of consumption. White meats were less frequently consumed by individuals who were older, who may benefit from practical ease, and red and processed meats were more frequently consumed by those who have fewer disabilities, who may do more of their own cooking and shopping, and by those who live with others compared to living alone, and so may more likely eat in company. 

For the dairy products, yoghurts, custards, and blancmanges were consumed most frequently by the sample as a whole and were more frequently consumed by females and those with a higher liking for or appreciation of the taste of dairy products. Soft cheeses were not frequently consumed, but were consumed more frequently by those wearing fewer dentures and by those with a higher liking for dairy products, those who were less concerned by physical abilities, those who were less affected by habit and those who were more affected by medical concerns. Hard cheeses were more frequently consumed by males and those who had more years of education, and those with a higher liking for or appreciation of the taste of dairy products. 

With a focus on the barriers and facilitators, softer dairy product consumption was associated with a higher liking for or a higher appreciation of the taste of dairy products. Liking and taste are strong predictors of food intake in older adults, as discussed above, and effects for dairy products specifically have previously been elucidated [25,26,57,58,59]. An appreciation for taste may be particularly prominent for this product group, furthermore, given the variety of tastes within the dairy category, from salty, smoked, and blue cheeses to sweet, flavoured yoghurts and desserts, and the variety in taste intensity. Strategies to increase liking that focus on flavour enhancement or addition may be less suitable for softer dairy products, while strategies that focus more on experiencing and embracing the variety of tastes that naturally exist may be more advisable. Previous work demonstrates the value of tastings for increasing product acceptance [59], and larger food repertoires have been associated with improved health. The increased consumption in our data, furthermore, by those with less reliance on habit, would suggest an openness to differing tastes and flavours in softer dairy consumption. 

The associations with perceived ability most likely reflect the ease with which softer dairy products can be consumed, not only due to softer textures but also due to the lack of cooking required, the individual packaging, and the light-weight nature of many of these foods [25,59]. These findings suggest that focusing on increasing the convenience of consuming these foods, again in terms of ease and effort, may be of value. Consumption in those with greater medical concerns may also reflect the findings based on perceived ability or maybe a reflection that those with medical concerns that are traditionally associated with diet, such as high cholesterol and high body-weight are or were often advised to steer away from hard cheeses due to their high fat and salt content and to replace these with softer dairy options [57]. The evidence is currently suggesting that this avoidance is likely unnecessary and that all dairy products can contribute to good health [21,22,23,57], but it will take time for consumers to gain this advice and act accordingly. 

Consideration of the demographic and lifestyle factors offers limited added understanding for encouraging softer dairy consumption. Some benefits may be gained by specifically targeting males, those more able or with better dentition, and those with more education, but health and environmental benefits will be gained from increased consumption of more sustainable choices by the whole population. 

Taken together, the data presented suggest that taste, enjoyment, convenience, or low required effort are key determinants of the consumption of the more sustainable meat and dairy options considered. The importance of enjoyment, taste, and convenience also emerged in our earlier analyses of the barriers to and facilitators of the consumption of a wider range of animal-based protein-rich foods in this population group, including fish and egg consumption. Both fish and egg consumption have a lower environmental impact than the consumption of red meats [14,15] and can provide considerable health benefits as a result of the provision not only of high-quality protein but also of essential fats and related micronutrients [60,61,62,63]. Both fish and egg consumption were also associated in our earlier analyses with lower concerns over spoilage and waste [25], as was also found here for white meat consumption specifically. This comparison is of interest, and arguably, the majority of dairy consumption can result in limited waste as a result of consumption of the entire product, assuming appropriate portion sizes are purchased. Eggs, however, may offer an additional advantage compared to fish, white meats, and dairy products, considering the lower cost and longer shelf-life of this protein-rich food [61]. 

Our findings suggest that enhancing or promoting the enjoyment, taste, convenience, and low waste of the more sustainable animal-based protein-rich foods may be of value for encouraging the consumption of these foods in older adults. There may be some opportunities here for the food industry in product development, product packaging, and marketing, e.g., in the development of tasty, ready-to-eat, healthy, and sustainable meals [30] or in the packaging and marketing of single portions. There are also opportunities for public health interventions, such as the provision of advice for older adults to make food consumption less effortful or to reduce waste, e.g., through the use of a freezer, through the provision of recipes to enhance taste, flavour and enjoyment, and the encouragement of cooking classes and clubs. We recently used a recipe intervention to increase egg consumption in older adults [64], and others have demonstrated benefits from cooking classes [65,66]. 

Our study is limited by the use of a dataset gained from an existing questionnaire. While the use of existing data can be considered a design strength, by gaining added value from existing data and by reducing the participant burden and research costs necessary for obtaining a new dataset, the development work for the questionnaire was undertaken over ten years ago [26], and some barriers to and facilitators of consumption will change over time. Notably, sustainability concerns may have been less prominent ten years ago, and while some of these concerns may be associated with food provenance, quality and freshness, and with farming and animal welfare, wider environmental concerns were absent from our initial focus group discussions [26]. Attitudes and concerns in the older population group, however, are also likely to be slow to change [38]. The questionnaire also didn’t ask about barriers and facilitators to the consumption of the different meat or dairy products specifically, so some information may have been lost. The analysis, however, does find differences in the barriers and facilitators between these products, and suggestions for change can be made. Limitations in the original dataset also remain; study of a likely healthy volunteer sample, use of self-report measures, use of a simple FFQ, and failure to consider all factors that may affect intakes in older adults, including health conditions, medication taking and physical activity levels [25]. We can also make no comment on the likely impact of the strategies suggested or the value of these compared to other strategies. Further research to test our strategies would clearly be of value. 

## 5. Conclusions

This work aimed to understand the consumption of more sustainable animal-based protein-rich foods in older adults. White meat consumption was associated with fewer concerns over spoilage and waste, and stronger perceptions that meat is convenient, while red and processed meat consumption were positively associated with liking/taste, appearance and convenience. Yoghurt and soft cheese consumption were positively associated with liking/taste and medical concerns, and fewer concerns over abilities and habit, while hard cheese consumption was only associated with liking/taste. Taken together, these data suggest that enhancing or promoting the enjoyment, taste, visual appeal, ease-of-consumption, and low waste of the more sustainable meat and dairy options may be of value for encouraging the consumption of these foods in older adults.

## Figures and Tables

**Table 1:** Results of all regression analyses for meat consumption (N = 340).

	White Meats		Red Meats		Processed Meats	
Regression Equations for the Final Models	R = 0.26, R^2^ = 0.07, adj. R^2^ = 0.06, F(4,333) = 6.11, *p* < 0.001		R = 0.32, R^2^ = 0.11, adj. R^2^ = 0.10, F(3,333) = 12.90, *p* < 0.001		R = 0.34, R^2^ = 0.12, adj. R^2^ = 0.10, F(5,333) = 8.58, *p* < 0.001	
	Beta	*p*	Beta	*p*	Beta	*p*
Gender (male/female)	0.257	0.93	**0.112**	**0.04**	0.324	0.89
Age (years)	**−0.167**	**<0.01**	0.397	0.93	0.152	0.93
Living status (alone/with others)	0.847	0.95	0.141	0.99	**0.142**	**<0.01**
Area of residence (South/Midlands and Wales/North England/Scotland, and Northern Ireland)	0.278	0.97	0.259	0.99	0.442	0.98
Multiple Index of Deprivation (0–1)	0.092	0.95	0.777	0.99	0.256	0.96
Years of education (years)	**−0.129**	**0.02**	0.958	0.97	0.970	0.98
Body Mass Index (kg/m^2^)	0.633	0.98	0.240	0.98	0.624	0.98
Denture wearing (0/0.5/1)	0.387	0.96	0.659	0.97	0.556	0.99
Physical disabilities (0/0.33/0.66/1)	0.364	0.89	**−0.120**	**0.02**	0.256	0.99
Receive help/food delivered (0–1)	0.353	0.96	0.227	0.73	0.629	0.96
Eating out (0–1)	0.499	0.98	0.920	0.95	0.329	0.94
Season of completion (summer/winter)	0.345	0.98	0.957	0.99	0.862	0.99
Liking	0.780	0.89	**0.294**	**<0.001**	**0.166**	**<0.01**
Healthiness	0.289	0.93	0.080	0.73	0.246	0.71
Texture	0.494	0.86	0.200	0.89	0.182	0.79
Appearance	0.494	0.87	0.540	0.94	**0.117**	**0.03**
Affordability	0.147	0.95	0.051	0.91	0.559	0.88
Freshness	0.750	0.97	0.660	0.94	0.189	0.73
Quality	0.611	0.96	0.360	0.93	0.658	0.66
Origins	0.607	0.95	0.555	0.98	**−0.160**	**<0.01**
Spoilage	**−0.156**	**<0.01**	0.799	0.95	0.232	0.82
Cooking for myself	0.797	0.79	0.564	0.90	0.469	0.74
Availability	0.415	0.87	0.816	0.94	0.586	0.87
Convenient	**0.135**	**0.01**	0.557	0.89	**0.123**	**0.03**
Able	0.131	0.87	0.379	0.60	0.788	0.92
Effort	0.253	0.99	0.372	0.97	0.997	0.99
Habit	0.068	0.96	0.837	0.91	0.472	0.86
Medical	0.542	0.90	0.168	0.92	0.503	0.87
Media reports	0.228	0.91	0.588	0.87	0.705	0.86

Significant associations (*p* < 0.05) are emboldened.

**Table 2:** Results of all regression analyses for dairy consumption (N = 347).

	Yoghurt		Soft Cheeses		Hard Cheeses	
Regression Equations for the Final Models	R = 0.23, R^2^ = 0.05, adj. R^2^ = 0.05, F(2,332) = 9.51, *p* < 0.001		R = 0.30, R^2^ = 0.09, adj. R^2^ = 0.07, F(5,332) = 6.24, *p* < 0.001		R = 0.24, R^2^ = 0.06, adj. R^2^ = 0.05, F(3,332) = 6.71, *p* < 0.001	
	Beta	*p*	Beta	*p*	Beta	*p*
Gender (male/female)	**0.205**	**<0.001**	0.407	0.98	**−0.111**	**0.04**
Age (years)	0.813	0.98	0.894	0.93	0.160	0.97
Living status (alone/with others)	0.488	0.97	0.755	0.99	0.243	0.97
Area of residence (South/Midlands and Wales/North England/Scotland, and Northern Ireland)	0.463	0.99	0.155	0.99	0.179	0.99
Multiple Index of Deprivation (0–1)	0.331	0.99	0.091	0.98	0.637	0.96
Years of education (years)	0.176	0.97	0.625	0.96	**0.127**	**0.02**
Body Mass Index (kg/m^2^)	0.653	0.99	0.703	0.99	0.545	0.99
Denture wearing (0/0.5/1)	0.169	0.99	**−0.117**	**0.03**	0.719	0.98
Physical disabilities (0/0.33/0.66/1)	0.960	0.96	0.978	0.67	0.558	0.96
Receive help/food delivered (0–1)	0.478	0.99	0.473	0.85	0.619	0.98
Eating out (0–1)	0.998	0.97	0.577	0.99	0.263	0.96
Season of completion (summer/winter)	0.100	0.99	0.573	0.99	0.091	0.98
Liking	**0.106**	**0.05**	**0.214**	**<0.001**	**0.153**	**0.005**
Healthiness	0.477	0.74	0.828	0.71	0.880	0.73
Texture	0.311	0.91	0.313	0.71	0.925	0.91
Affordability	0.695	0.97	0.177	0.93	0.119	0.97
Quality	0.619	0.95	0.484	0.95	0.124	0.95
Origins	0.442	0.98	0.328	0.97	0.379	0.98
Spoilage	0.233	0.98	0.445	0.91	0.470	0.97
Single	0.252	0.94	0.686	0.80	0.879	0.94
Availability	0.132	0.97	0.403	0.80	0.119	0.97
Convenient	0.932	0.87	0.788	0.82	0.769	0.87
Able	0.844	0.99	**−0.111**	**0.047**	0.246	0.98
Effort	0.434	0.99	0.855	0.81	0.562	0.98
Habit	0.385	0.88	**−0.119**	**0.04**	0.619	0.88
Medical	0.757	0.98	**0.126**	**0.03**	0.341	0.98
Media reports	0.477	0.91	0.214	0.77	0.879	0.91

Significant associations (*p* < 0.05) are emboldened.

## Data Availability

The data presented in this study are available in BORDaR, the Bournemouth Online Data Repository for Bournemouth University, UK, and on request from the corresponding author.
